# Exploitation of Unique Properties of Zeolites in the Development of Gas Sensors

**DOI:** 10.3390/s120405170

**Published:** 2012-04-20

**Authors:** Yangong Zheng, Xiaogan Li, Prabir K. Dutta

**Affiliations:** 1 School of Electronic Science and Technology, Dalian University of Technology, Dalian 116023, China; 2 Department of Chemistry, the Ohio State University, 100 W. 18th Ave., Columbus, OH 43210, USA

**Keywords:** biomedical, combustion, environmental, metal oxides, harsh environment

## Abstract

The unique properties of microporous zeolites, including ion-exchange properties, adsorption, molecular sieving, catalysis, conductivity have been exploited in improving the performance of gas sensors. Zeolites have been employed as physical and chemical filters to improve the sensitivity and selectivity of gas sensors. In addition, direct interaction of gas molecules with the extraframework cations in the nanoconfined space of zeolites has been explored as a basis for developing new impedance-type gas/vapor sensors. In this review, we summarize how these properties of zeolites have been used to develop new sensing paradigms. There is a considerable breadth of transduction processes that have been used for zeolite incorporated sensors, including frequency measurements, optical and the entire gamut of electrochemical measurements. It is clear from the published literature that zeolites provide a route to enhance sensor performance, and it is expected that commercial manifestation of some of the approaches discussed here will take place. The future of zeolite-based sensors will continue to exploit its unique properties and use of other microporous frameworks, including metal organic frameworks. Zeolite composites with electronic materials, including metals will lead to new paradigms in sensing. Use of nano-sized zeolite crystals and zeolite membranes will enhance sensor properties and make possible new routes of miniaturized sensors.

## 1. Introduction

**Figure 1.**
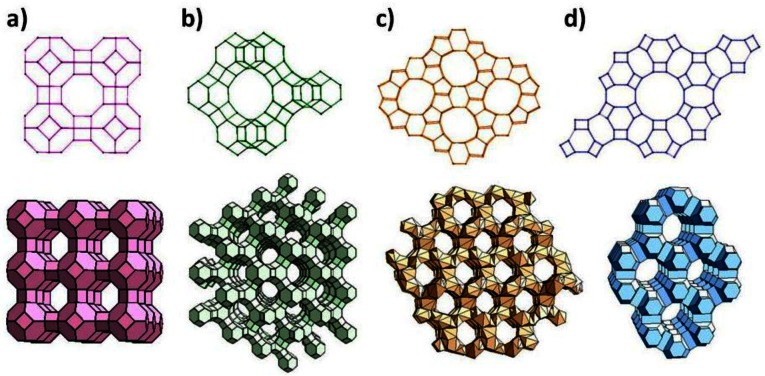
Representative zeolite frameworks, (with pore openings). (**a**) zeolite A (3D, 4.2 Å); (**b**) zeolite Y (3D, 7.4 Å); (**c**) Zeolite L (1D, 7.1 Å); (**d**) ZSM-5 (silicalite) (2D, 5.3 × 5.6 Å, 5.1 × 5.5 Å) D—dimensions of channel system.

Zeolites are crystalline aluminosilicates with pores and channels of molecular dimensions [1–6]. Zeolites can be synthesized with different chemical compositions and distinct framework topologies, and about 170 of such topologies have been reported. Figure 1 shows the framework structure of four commonly used zeolites: zeolite A, zeolite Y, zeolite L and ZSM-5. Due to their ion-exchange properties, as well as adsorption and reactions of molecules within its cages, zeolites have found use in numerous applications in catalysis and separations. Use of the novel properties of zeolites for sensor applications is of more recent vintage, yet, several recent reviews have summarized the role of zeolites in sensor development [7–9]. In this review, we primarily focus on the research in the past decade, and approach this review from the unique properties of the zeolite.

There are several physical and structural features of zeolites that have been exploited for sensing. The versatility of these materials is evident from the fact that the zeolite can be used to improve the performance of an existing sensor, as well as zeolites have been used as the sensing medium. We briefly discuss these properties, followed by examples detailing their use in sensor development.

The internal microporosity of zeolites that gives rise to the high surface area also provides sites for adsorption of molecules. Upon adsorption, mass changes as well as optical properties are altered, which have been used for the sensor transduction. Selectivity towards analytes has also been observed by adsorption of species within a zeolite. Microporous spaces within the zeolite also serve as a host for guest species. Optical/electrical properties change of the guest species upon interaction with gases has been used for sensing.

Zeolites are often referred to as molecular sieves, since the crystalline nature allows for strict size discrimination. For example, *n*-butane can enter through the pore openings of calcium-exchanged zeolite A (Figure 1(a)), but its isomer, isobutane cannot. Also, if several molecules can enter the zeolite, their intrazeolitic diffusion can vary due to both molecule-zeolite interactions and intermolecular interactions, and thus transport through the zeolite can be quite different for these molecules. Such diffusional discrimination has been exploited in developing filters, which when placed prior to the sensing device provide selectivity.

Zeolites can be modified to incorporate strong acidic functionality as well as can serve as host to various metals and metal oxides. Such systems are at the heart of zeolite catalysis, and find extensive applications in the chemical and petrochemical industry [1–6]. Selective transformation of molecules via zeolite catalysis has been exploited for promoting selectivity and sensitivity of electrochemical/optical/calorimetric sensors, with the active zeolite acting as a filter through which the gases pass prior to reaching the sensor.

The negative aluminosilicate framework of zeolites necessitates the presence of neutralizing ion-exchangeable cations within the framework. These cations can influence adsorption, diffusion and catalytic properties of zeolites, and thereby influence sensing behavior. The Si/Al ratio of the framework controls the number of cations and the hydrophilicty of the zeolite, and has been used for selective adsorption based on polarity of the analyte.

Also, the extraframework cations are bound electrostatically at preferential sites and can perform energy activated motion between these sites. Presence of guest molecule within the zeolite can interfere with this motion and has been used as the basis of sensing. Interaction of the molecule with the cation is manifested in the change in impedance/capacitance as measured by the frequency dependent impedance spectra. With this introduction, we address specific applications, focusing on how the above-described physical and chemical properties of zeolites have informed sensing paradigms.

## 2. Adsorption of Molecules in Zeolites

**Figure 2.**
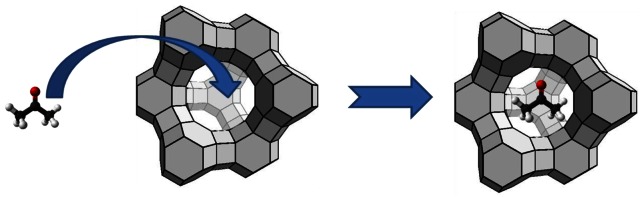
Adsorption of an acetone molecule into a zeolite Y supercage (window 7.4 Å, cage 13.2 Å).

Figure 2 shows a conceptual diagram of adsorption of an acetone molecule into the supercages of zeolite Y. Such adsorption will result in a mass change, and this capacity of the zeolite to adsorb gas molecules has been exploited in developing gas sensors.

### 2.1. Frequency Dependent Measurements

Mass-sensitive transducers such as surface acoustic wave (SAW), quartz crystal microbalance (QCM) and microcantilevers have employed the zeolite layer as a functional element. When the zeolite layer adsorbs the gas molecules from the surrounding, the mass changes (Δm) per unit surface/volume results in a proportional frequency shift (Δf), which can be measured:

$$\frac{\Delta {\text{f}}^{2}}{f^{2}}\propto \frac{\Delta m}{m}$$

where f is fundamental resonance frequency, m is the total mass of sensing layer.

Zeolite films were grown on piezoelectric sensor devices (quartz crystal microbalance, QCM) via seeding of nanocrystals followed by secondary growth. Both zeolite A and zeolite beta of thicknesses <1,000 nm have been grown on QCM [10,11]. These devices functioned as humidity sensors. Sensors based on zeolite beta exhibited interference towards pentane, hexane and cyclohexane, since the 7.7 Å pore size allowed adsorption of these molecules into the zeolite. Regarding quantitative aspects of the sensor performance, exposure to 150 ppm water led to a frequency shift of 2,000 and 400 Hz for the zeolite A and zeolite beta coated QCM devices, respectively. The response times for a 40 ppm concentration step were ∼90 and ∼60 s for zeolite A and zeolite beta QCM devices, respectively. The recovery times were faster for the zeolite beta QCM device, which was explained as arising from the larger pore channel (7.7 Å, water molecule is 2.65 Å) and lower hydrophilicity due to the higher Si/Al ratio. In a more detailed study with zeolite A (LTA), it was found that thinner films of zeolite A (65, 470 nm) performed better than thicker films (980 nm), with 80 ppm water causing a mass increase of 0.075 μg of sorbate/μg of film [11].

**Figure 3.**
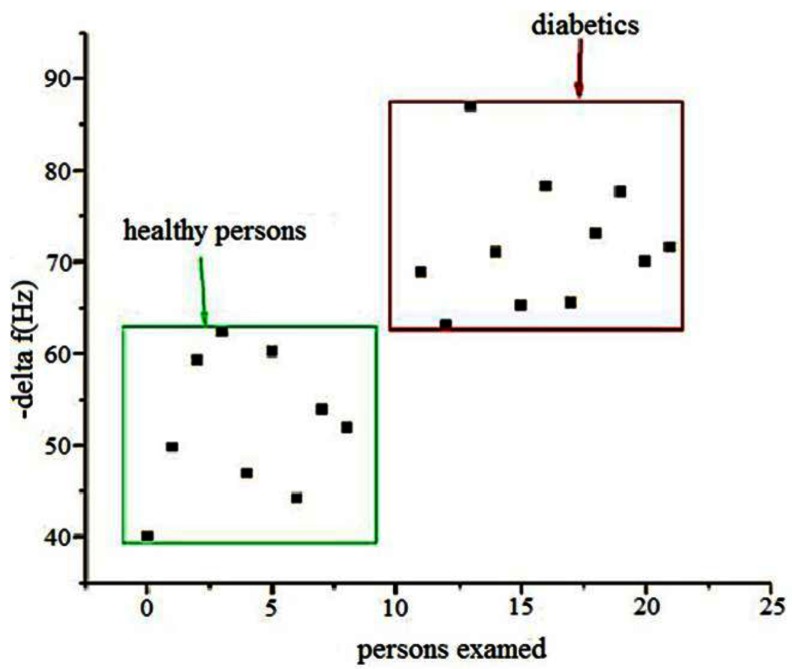
Breath analysis comparisons of healthy persons and diabetics using Ag-ZSM5 on a QCM device. (Adapted from Reference [12]).

Acetone in breath can serve as a diagnostic for diabetes, and using Ag^+^-ZSM-5 on QCM, a detection limit of 0.26 ppm of acetone with a response time of 90 s was reported [12]. The selection of Ag^+^ was made to increase the polarity of the ZSM-5 to increase interaction with acetone, though the basis for this hypothesis was not proven. The Ag^+^-ZSM-5-QCM device also responded to ethanol, but with only 25% of the signal relative to acetone. Humidity was a major interferent and needs to be absent from the analyte gas stream. Figure 3 shows the results with breath analysis of healthy *versus* diabetic patients, with the y-axis representing the change in frequency as compared to dry N_2_ as baseline. This device could distinguish between diabetic and healthy persons based on acetone in their breath. Though the diabetic patients clustered together (with 0.26–4.9 ppm acetone in breath), the study pointed out that the correlation of acetone in breath with blood sugar is not yet established.

Microcantilevers vibrate at a specific frequency when excited by an ac voltage. Adsorption of freon in ZSM-5 deposited on a microcantilever led to a sensitivity of ±0.0024% with a minimum mass detection of 3.5 × 10^−9^ g. The freon gas sensor did not exhibit interference to ethanol [13].

With Co-zeolite β/cantilever, nitrotoluene could be detected at concentration of 1 ppmv [14]. The cantilevers were produced by a five-step photolithography procedure involving initial formation of the strain gauges and the electrical contacts, followed by the formation of the cantilevers. Paddle shaped cantilevers provided more area for zeolite deposition. Cobalt exchanged zeolites were chosen because of the improved interaction between nitrotoluene and cobalt; with toluene, the signal was 90 times lower than nitrotoluene. The limitation in sensitivity was from the baseline noise (9 MHz), which can be controlled by the cantilever design, and the best detection limit was 0.2 ppmv.

Zeolite Y grown hydrothermally on a Metglas Magnetoelastic strip led to a composite that could sense CO_2_ in a nitrogen atmosphere [15]. Magnetoelastic materials resonate at a specific frequency (f_o_) in the presence of an alternating current magnetic field. The f_o_ for zeolite-metglas composite changed from 105.80 kHz in pure CO_2_ to 107.5 kHz in N_2_, because of the higher adsorption capacity of the zeolite for CO_2_. The minimum detectable CO_2_ concentration was reported as 0.33 vol% CO_2_, but the device exhibited interference to humidity at concentrations greater that 2% relative humidity. The sensor was reported to be insensitive to methane, and appeared to be stable over a period of month.

### 2.2. Optical Measurements

Adsorption of analytes in a zeolite is a way to preconcentrate the analyte. This strategy was exploited to enhance the infrared spectral signal of *n*-hexane by a factor of 180 by adsorption in silicalite-1 coated on a ATR (attenuated total reflection) element [16]. Both ZnS and Si ATR substrates were examined, with comparable results (ZnS coated with zeolite on both sides), the response time (monitoring the 2,960 cm^−1^ band of hexane) was 250 s (p/p_o_ change from 0 to 0.01 at 27 °C). No data was provided on the recovery times, though it was suggested that desorption will require higher temperatures, which makes it somewhat impractical for field use.

Adsorption can also cause change in other optical properties, for example, in the refractive index, and has been exploited in development of sensors for detecting trace organic vapors [17]. Silicalite was coated on a long period fiber grating (LPFG). The grating is prepared by inscribing a periodic index perturbation into the core of an optical fiber with a laser (e.g., with CO_2_ laser with a period of 520 μm and a total grating length of 50 cm). There are certain resonant wavelengths (λ_R_) that couple the core and cladding modes when propagating through the fiber, and any change of the refractive index of the surrounding medium will alter λ_R_, e.g., absorption of 16.5 ppm isopropanol in the zeolite layer led to λ_R_ shift from 1,558 to 1,556.5 nm under ambient conditions. Signals for 22 ppb toluene were observed with 25–45 min response time. Molecules such as isopropanol exhibited a faster response than toluene due to their smaller kinetic size, thereby promoting rapid equilibration within the zeolite. Recovery of the device after sensing measurements with toluene required purging with air at 350 °C, and this need for heat during recovery will add complication in device fabrication.

### 2.3. Conductivity of Conducting Polymer-Zeolite Composites

The adsorption properties of zeolites have been exploited in conducting polymer-zeolite composites to promote selectivity [18–21]. Polyaniline has been the most investigated polymer, with zeolite A composites exhibiting high selectivity to CO, the Ca^2+^-form of the zeolite being most effective. Composites of polyaniline with zeolite A(K^+^) and polyaniline-polyamide 6 with zeolite A were found to have different sensitivity to the components of lacquer thinner (acetone, methylether ketone, methanol and toluene) depending on whether the composites were prepared in pellet (sensitive), film (sensitive) or electrospun fiber bundles (insensitive) [20]. It was noted that high methanol sensitivity is related to its solvent properties towards the polyamide, though the exact role of the zeolite in promoting selectivity towards methanol is not clear. A major interferent for the conducting polymer-based sensors is humidity.

## 3. Diffusion Discrimination of Analytes Based on Zeolite Topology

By exploiting the diffusion characteristics of different gases through zeolites, selectivity of sensors can be improved. Many reports are available, especially with electrochemical sensors that are covered with a zeolite layer.

### 3.1. Conductivity Measurements

**Figure 4.**
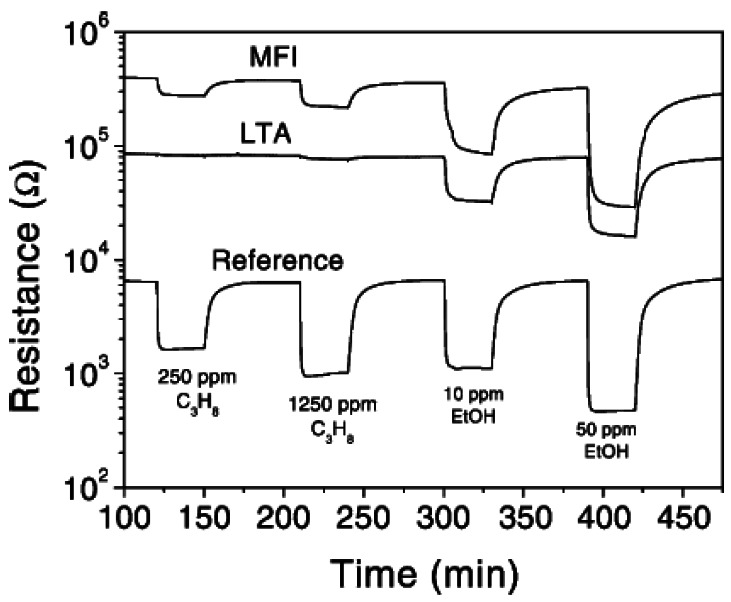
Comparison of response between Pd-SnO_2_ sensors coated with silicalite, zeolite A and uncoated for propane and ethanol, showing selectivity to ethanol with zeolite A layer, 350 °C with 50% relative humidity (adapted from Reference [22]).

For semiconducting Pd-doped SnO_2_, silicalite and zeolite A layers on the SnO_2_ led to suppression of signal due to methane, propane, while the resistance change with ethanol remained unaltered, thereby discriminating between these molecules [22,23]. The influence of the zeolite layer on the sensor performance is demonstrated well in Figure 4. With zeolite A, there is no response to propane, even at concentration of ∼1,250 ppm, while still being responsive to ethanol. With silicalite, both propane and ethanol exhibit a signal. The explanation for the selectivity was that the propane (or methane) does reach the Pd-SnO_2_ passing through the zeolite, gets oxidized and the resulting water adsorbed in the hydrophilic LTA layer blocks the motion of the hydrocarbon gas, thus providing a selective ethanol sensor. Addition of moisture in the sensing stream provides additional blocking sites and can completely remove the interference from the hydrocarbon. Zeolite layers were grown hydrothermally (with complete coverage of the semiconductor surface) or just coated from a suspension, the latter being a more convenient fabrication step, since the semiconducting oxide was not exposed to hydrothermal conditions and response times were also better with the non-continuous zeolite film.

Semiconducting WO_3_ was overlaid with hydrogen forms of four zeolites (zeolite A, NH_4_ ZSM-5, H-beta and H-zeolite Y) and tests with varying concentrations of NO_2_, CO and ethanol exhibited different resistance change patterns, and it was suggested that the composite zeolite-metal oxide sensors could be the basis for a sensor array device [24]. For NO_2_, H-zeolite Y layer led to the doubling of the sensor response as compared to WO_3_ (204 ppb NO_2_), though in the presence of water, this improvement in sensitivity disappeared. The effect of the zeolite layer typically led to increased recovery times as compared to the base WO_3_.

Ethanol and isopropanol could be discriminated with a zeolite-WO_3_ and zeolite-chromium titanium oxide semiconducting sensor [25]. Acid forms of zeolites A, ZSM-5 and zeolite Y were examined. Both diffusive and catalytic aspects of the zeolite were relevant to the sensing, though the catalysis chemistry related to the alcohols was not evaluated. The enhanced response to ethanol over isopropyl alcohol (IPA) with zeolite A layer was proposed to arise from a molecular sieving effect due to the larger size of IPA. Computer modeling of diffusion process indicated that the discrimination between these molecules arises from size and shape selectivity imposed by the zeolite layer.

**Figure 5.**
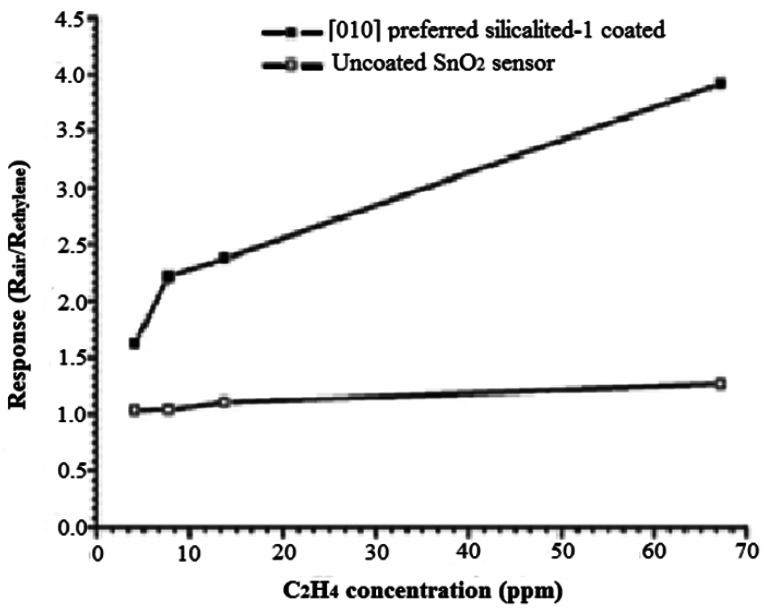
The response of preferentially coated [010] silicalite-1 layer and uncoated SnO_2_ thin film sensors towards ethylene, 350 °C (adapted from Reference [26]).

An excellent example of the improved selectivity by employing zeolite layer is provided by silicalite-1 on SnO_2_ for detecting ethylene in the presence of water [26]. Also, by hydrothermally growing [010] oriented silicalite-1 on the SnO_2_, the response and recovery times (14 s, 144 s) were improved as compared to randomly distributed (25 s, 208 s) silicalite-1 on the SnO_2_ surface. Figure 5 compares the ethylene response for SnO_2_ and SnO_2_ covered with the [010] preferred-orientation silicalite-1 layer. Improved sensitivity was proposed to arise from preferential adsorption of ethylene into the silicalite-1, as well as the fact that the oriented silicalite-1 preconcentrates the ethylene into its vertical channels.

**Figure 6.**
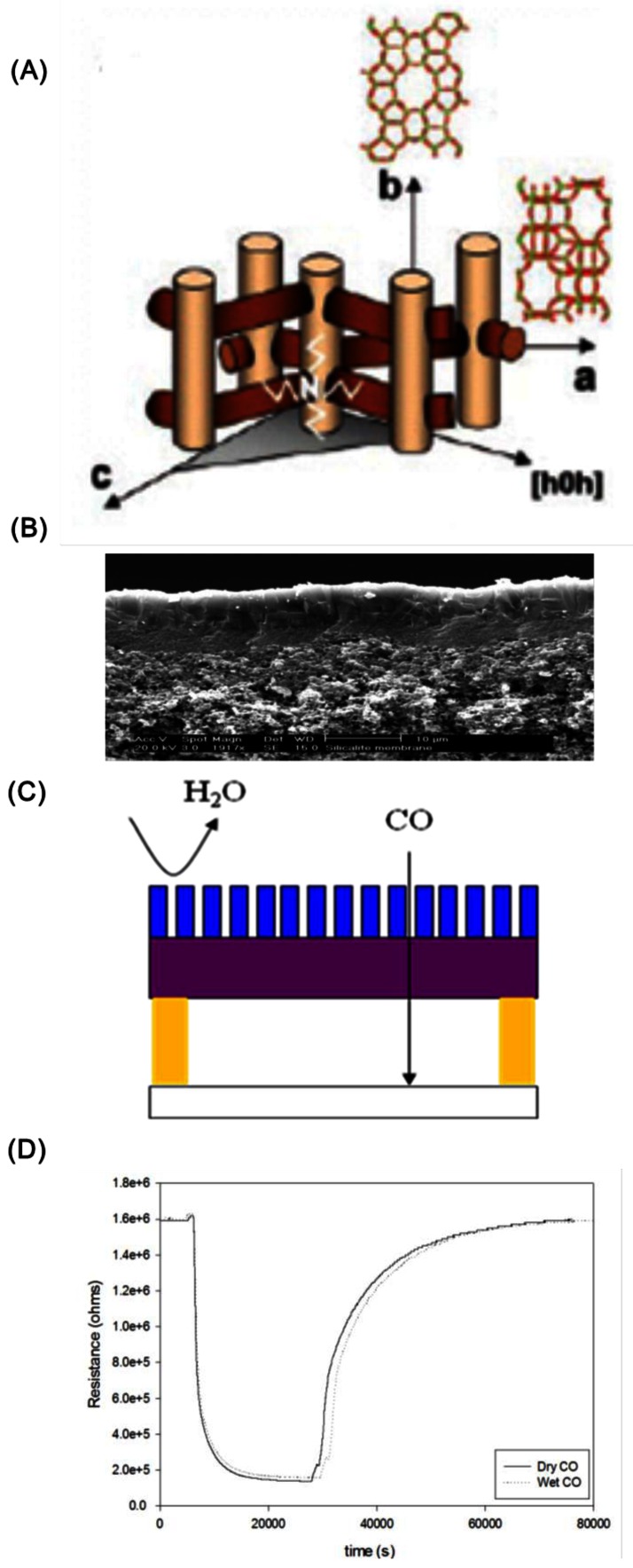
Strategy for rejection of water with a hydrophobic silicalite zeolite filter. (**A**) Channel system of silicalite; (**B**) SEM of zeolite layer on alumina; (**C**) zeolite layer spatially separated from the titania sensor; (**D**) response of the titania sensor with dry and wet CO, 600 °C (adapted from Reference [27]).

Figure 6 shows an example where the zeolite (silicalite, Figure 6(A)) was grown on an alumina membrane (Figure 6(B), SEM micrograph of cross section) and the strategy of water rejection was based on the scheme shown in Figure 6(C) [27]. Rejection of water in a moist CO stream was made possible by the hydrophobicity of the zeolite silicalite layer, ensuring that only CO was reaching the semiconductor (titania) surface. Figure 6(D) shows the sensor (titania) response for dry and wet CO, and the signals were similar. However, the response/recovery times were too long (hours) in the presence of the zeolite and therefore, somewhat impractical.

### 3.2. Amperometric Measurements

An amperometric sensor made with a zeolite A coating on a Pt-yttria stabilized zirconia used as sensing and reference electrodes (zeolite coating on only one electrode) and yttria stabilized zirconia as electrolyte exhibited response to both O_2_ and CO_2_ with a bias voltage of 1.8 V [28]. Upon coating the device with a continuous zeolite film, the signal for both O_2_ and CO_2_ decreased, but more so for CO_2_. It was suggested that the preferable diffusion of O_2_ through zeolite led to this effect. The preferential selectivity of oxygen over CO_2_ could not be directly correlated to molecular sieving (similar sizes of analytes), but diffusional aspects of the zeolite film were considered important.

## 4. Zeolite-Induced Catalysis Promoted Selectivity

There are many examples where the catalytic properties of zeolites have been exploited to promote sensor performance, with a variety of gas-zeolite interactions towards this goal, as explained below.

### 4.1. Conductivity Measurements

Acid sites in zeolite promote cracking reaction of *n*-alkanes. Using catalytic Cr-zeolite Y and Cr-zeolite beta film coated on semiconducting chromium titanium oxide, the cracking patterns and shape and size effects were found to improve selectivity to linear alkanes [29,30]. Two zeolite layers were examined, Cr-zeolite Y and Mo-zeolite Y, with linear alkanes (C_7_-C_10_) as analytes. Chromatographic (GC-MS) studies showed that over Cr-zeolite Y, heptanes was unreactive, nonane completely reacts making methylpropanol and water, whereas octane and decane produce small linear and branched chain alkanes (<C_6_). In keeping with these results, the nonane produced a response with chromium titanium oxide that was distinct from the other alkanes. With Mo-zeolite Y, the products were primarily water, aldehydes and ketones, with acetaldehyde as the primary product, indicating that cracking and partial oxidation of the *n*-alkanes is dominant. The sensing properties observed for the *n*-alkanes were arising from the reactivity of the reaction products on the chromium titanium dioxide, rather than the analytes themselves. We will see other examples below, where the analyte has been modified chemically and the resulting product used for the sensor response, establishing a new paradigm for sensing.

Pt-doped ZSM-5 as a cover layer on semiconducting SrTi_0.8_Fe_0.2_O_3-δ_ led to selective detection of propane with discrimination against H_2_, NO and CO [31]. Besides the zeolite, choice of proper electrode (e.g., Pt) was also important to promote selectivity. Interestingly, the zeolite cover suppressed the sensor response of the unsaturated hydrocarbon (propene) as compared to the saturated hydrocarbon (propane), though the reason for this was not clear, but possibly related to the catalytic activity of the zeolite.

Noble metals are often added to semiconducting metal oxides to promote selectivity and sensitivity for gas detection. At high temperatures of operation, there is the possibility of interaction of the metal with the sensing metal oxide, thereby altering its electrical properties. In order to avoid this interaction, the Pt was included in the supercages of zeolites Y, which was then mixed with the semiconducting oxide, TiO_2_. It was reported that the Pt-zeolite-TiO_2_ composite exhibited selectivity to propane and discriminated against CO. It was proposed that the resistance change of TiO_2_ was arising from interaction with water, the oxidation product of propane catalyzed by the embedded Pt zeolite within the TiO_2_ [32].

With WO_3_ as the sensing oxide layer, it was reported that the use of H-ZSM5 as an overlayer led to a 19 fold increase in sensor response to NO_2_ as compared to an unmodified sensor. Tests were also done with acetone, with the best results obtained for Cr-zeolite layer. It was proposed that the Cr-zeolite (ZSM-5, zeolite A) catalytically converted the acetone to a reaction product that was more sensitive on the WO_3_ sensor. The H-ZSM5 did not exhibit an increase in sensing response to acetone [33].

### 4.2. Potentiometric Measurements

**Figure 7.**
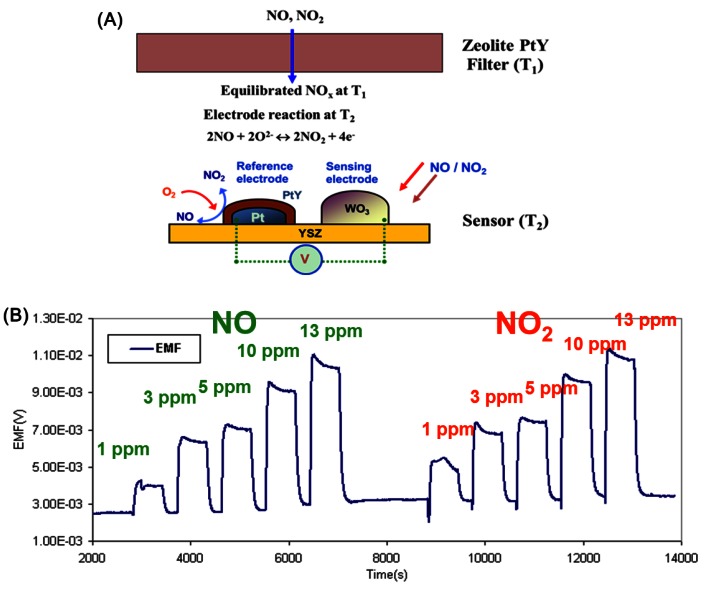
(**A**) Schematic for a total NO_x_ sensor, zeolite filter at 450 °C (T_1_), YSZ sensor with reference (Pt-zeolite Y) and sensing (WO_3_) electrode at 600 °C (T_2_); (**B**) Sensor response to NO (left) and NO_2_ (right) (adapted from Reference [36]).

In all these examples discussed, the zeolite layer is in contact with the active oxide sensing layer. It is likely that the sensing semiconducting surface is altered because of the interaction with the zeolite, especially at the high temperatures often necessary for sensing. Another drawback for such design lies in that the physiochemical filters have to work at the same temperature as the active sensing layers which can limit the performance of either the filters or the sensing layers, since both necessarily do not have the same optimum operating temperature. The concept of separating the zeolite layer from the sensor has led to a new paradigm in sensing. This is best demonstrated for detection of NO_x_, in a complex background of other gases. NO_x_ is primarily present as NO/NO_2_ and often the interest is in determining total NO_x_ (NO + NO_2_). There are many examples of potentiometric devices that can detect NO and NO_2_. However, since NO gets oxidized and NO_2_ gets reduced, their response are opposite, and mixtures of NO and NO_2_ will lead to cancellation of signal, making calibration impossible. Using a Pt-zeolite Y layer separated and ahead of a potentiometric (yttria stabilized zirconia (YSZ) as electrolyte) sensor (Figure 7(A)), the NO_x_ passing through the zeolite gets equilibrated to a particular NO + NO_2_ composition depending on the temperature of the zeolite layer. If this equilibrated NO_x_ mixture contacts the potentiometric sensor, then a response would be observed as long as the sensing device is at a different temperature (T_2_) than the zeolite layer (T_1_), and the signal is a measure of total NO_x_ (NO + NO_2_) [34–38].

**Figure 8.**
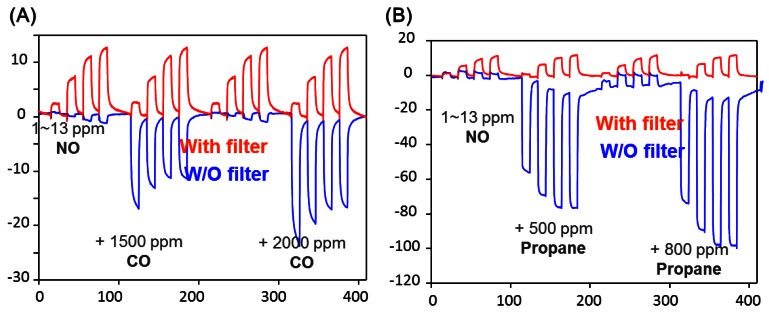
Interference effects for device shown in Figure 7. (**A**) CO with (top) and without filter; (**B**) propane with (top) and without filter (adapted from Reference [36]).

The active sensing unit YSZ is a mixed-potential type sensor working in accordance with an asymmetrical open circuit potential as the analyte reacts at the triple-phase-boundary with oxygen at each electrode. One of the electrodes is Pt covered with Pt-Na zeolite Y (reference) and the other one was covered with tungsten oxide (sensing) which was reported to exhibit a good sensitivity to NO. The reference Pt-Na zeolite Y equilibrates the NO and NO_2_. The results from such a device is shown in Figure 7(B), which shows that with the zeolite layer at 450 °C, the sensor at 600 °C, NO or NO_2_ provide similar signals. The use of the zeolite catalytic layer was also shown to reduce interference to CO, C_3_H_8_ and ammonia, gases expected to be interferents in combustion-based processes, such as lean burn engines. Figure 8 shows the data for 1–13 ppm NO with CO and propane in the presence and absence of the Pt-Na-zeolite Y filter. Stability tests of such sensors over a period of a week were reported.

A cross-cutting application of the above discussed NO_x_ sensors has been in human breath analysis for diagnosis of asthma [39]. Upper airway inflammation leads to increase of NO in breath from ∼5–10 ppb (normal) to levels as high as 100 ppb in diseased states. The NO_x_ sensor discussed above (Figure 7(A)) has sensitivities of ppm and would not be suitable for this breath application.

**Figure 9.**
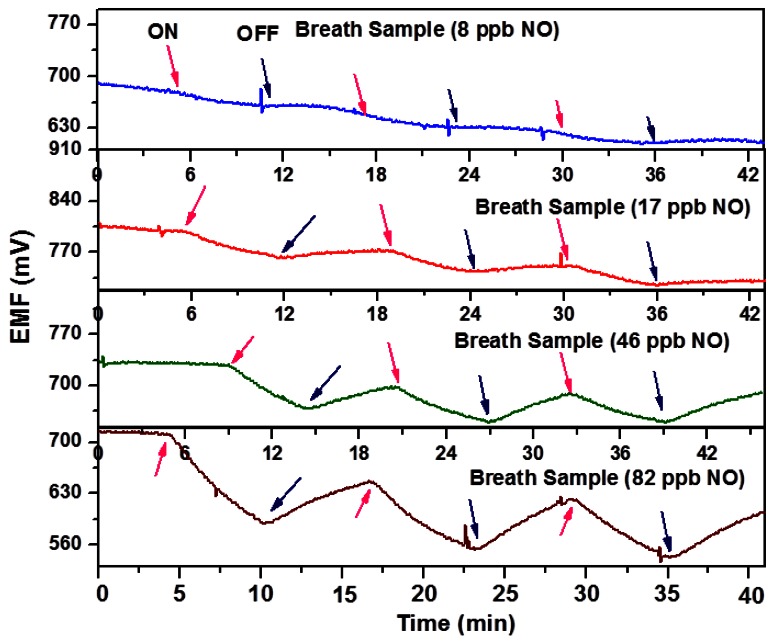
Response of a 20-sensor array using multiples of the unit device in Figure 7(A) towards ppb levels of NO in human breath (adapted from Reference [39]).

However, by stringing together 10–20 sensors in series, the sensitivity can be increased since the potentials are additive. Figure 9 shows the result from a 20-sensor array for human breath samples with NO concentration in the 8–82 ppb range, making it suitable for asthma applications. There are efforts to miniaturize these sensor arrays, with the goal of fabricating portable, hand-held asthma monitors [40].

In the zeolite-based potentiometric design [34–38], the zeolite and the sensor need to be spatially separated and at different temperatures, with higher differences in temperature leading to stronger NO_x_ signals. The basic reason for this increase in sensitivity is that with a larger temperature difference, there is a stronger driving force (higher ΔG) for equilibration. This imbalance in equilibration can also be accomplished by applying a voltage across the working/reference electrodes, and the ensuing chemical equilibration at the sensor leads to a measurable current. Using this strategy, an amperometric sensor has been reported [41].

**Figure 10.**
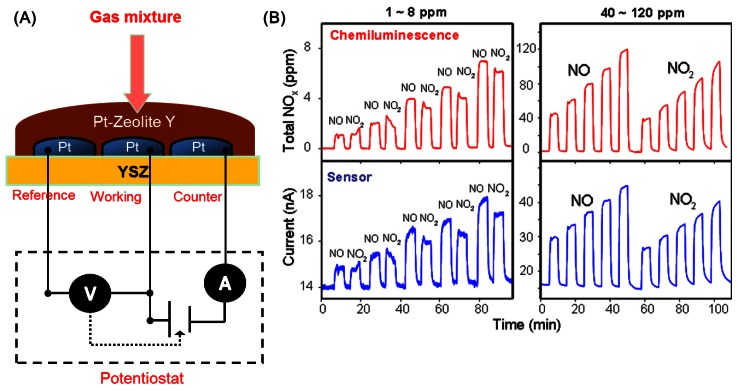
(**A**) Schematic of an amperometric sensor with a coating of Pt-zeolite Y; (**B**) comparison of the response of the sensor (bottom) with a chemiluminescence analyzer (top) towards NO and NO_2_ of varying concentrations (adapted from Reference [41]).

With the use of a zeolite layer covering the sensor, total NO_x_ (NO + NO_2_) can be measured, and interference to gases like CO and hydrocarbons are eliminated. Figure 10(A) shows a schematic of the sensor design, and Figure 10(B) compares the output of such an amperometric sensor with a chemiluminescence analyzer, clearly demonstrating the practicality of the device for ppm detection of total NO_x_. Stability tests for such sensors were reported over a 30-day period, and indicted that the background signal approached steady state after 15 days.

**Figure 11.**
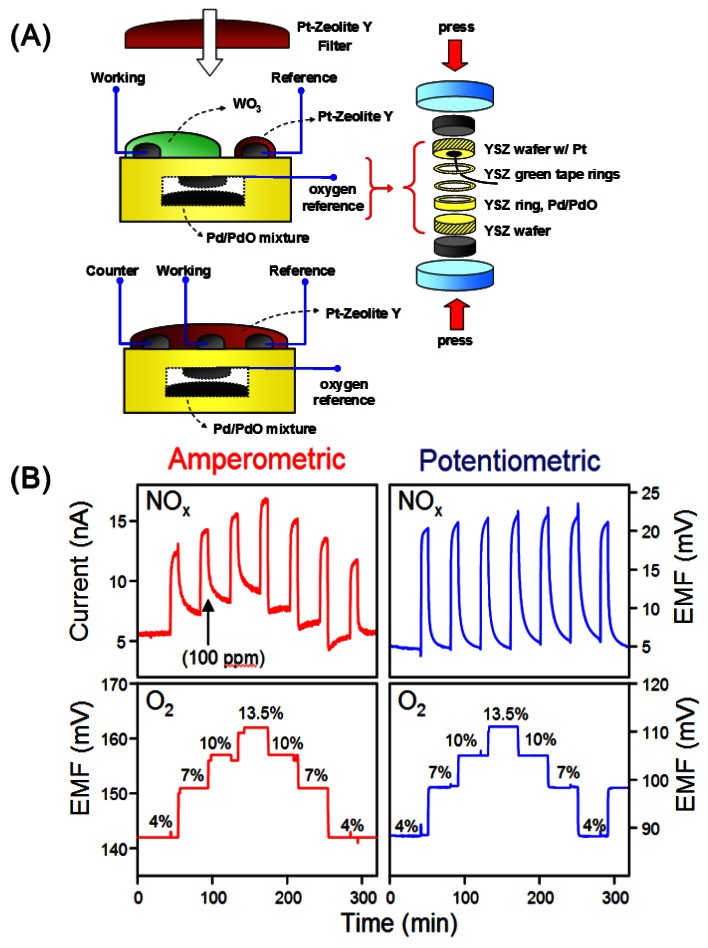
(**A**) A combined NO_x_-O_2_ sensor using potentiometric (top) and amperometric (bottom) detection; (**B**) Simultaneous measurement of NO and O_2_ (100 ppm NO with varying concentrations of O_2_) using the amperometric and potentiometric devices (adapted from Reference [42]).

The combination of the potentiometric and amperometric NO_x_ sensor just discussed with a reference-free O_2_ sensor has led to a bifunctional total NO_x_/O_2_ sensor, which measures NO_x_ and O_2_ simultaneously [42]. To the best of our knowledge, this is the only example where two analytes are being detected with an electrochemical sensor at the same time. The potentiometric based device exhibits lower interference to O_2_ as compared to the amperometric device, as shown in Figure 11. Stability tests over a period of 14 days was reported with satisfactory performance.

### 4.3. Optical Measurements

Catalytic reactions inside zeolite cages that lead to light emission have been developed as sensors. Examples include detection of acetaldehyde via reaction with O atoms in zeolite supercages, with discrimination against other aldehydes, such as formaldehyde, cinnamaldehyde, glutaraldehyde and benzaldehyde [43]. The selectivity was proposed to arise from the structural constraints imposed by the zeolite.

**Figure 12.**
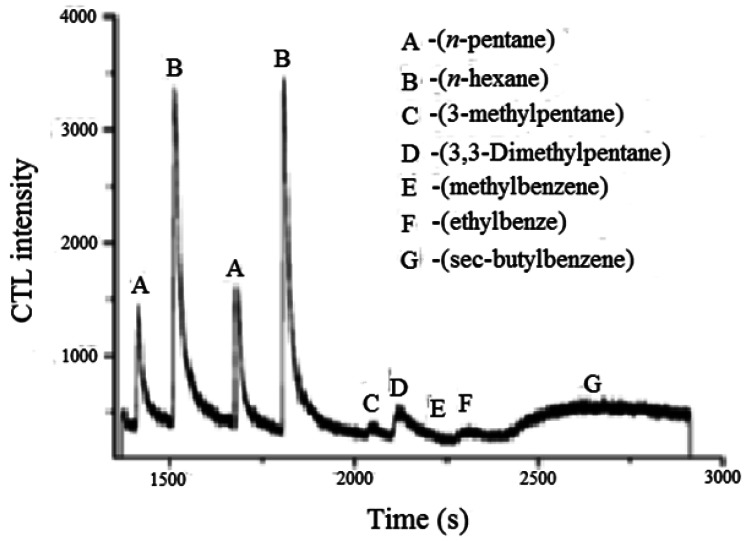
The intensity of cataluminescence for various alkanes, aromatics and alkyl aromatics on CsNaY-2 zeolite, 3.88 μg/mL of analytes, demonstrating sensitivity to n-alkanes (adapted from Reference [44]).

Using similar principles of light emission from an intrazeolitic catalytic reaction, a sensor for hexane has been reported [44]. The basicity of the zeolite was proposed to be important in the catalytic activity. Detection limit for *n*-hexane was found to be 0.55 μg/mL and minimal interference was noted from alcohols, other alkanes and aromatics. Figure 12 shows the cataluminescence data (measured at 460 nm) for a series of organic molecules (3.88 μg/mL) on CsNaY zeolite and demonstrates the selectivity for linear alkanes over branched alkanes and aromatics. A chemical reaction mechanism involving the formation of a four membered-ring transition state, involving an oxygen atom and carbonium ion was proposed.

### 4.4. Calorimetric Measurements

Heat liberated during a catalytic reaction on a zeolite has been coupled with a micromachined thin-film calorimeter for selective sensing of analytes [45]. With Pd-zeolite A layer, the sensor response was primarily to CO and discriminated against n- and i-butane, n-hexane and cyclohexane, whereas with a Pd-zeolite Y layer, the sensor response was still dominant with CO, but n-hexane and n-butane also exhibited a response. The explanation for the discrimination involved molecular sieving effects from the smaller pore zeolite A.

## 5. Host-Guest Chemistry with Zeolite as Host

There are reports of electrochemical and optical sensors that exploit the intrazeolitic space as a site for guest species which interact in specific ways with the analyte of interest.

### 5.1. Conductivity Measurements

By loading LiCl into stilbite with high and optimal dispersion, a conductive material was obtained that behaved as a humidity sensor with a dynamic range of 10^4^. The response times for adsorption and desorption were ∼26.5 and 38.6 s with humidity change from 0 to 63.2% [46].

In another study, C_60_ was deposited on zeolite Y films on a tantalum toothcomb type electrode, followed by thermal treatment to get the C_60_ into the zeolite cages [47]. The zeolite-C60 composites as well as potassium-C60-zeolite electrodes exhibited semiconductor characteristics. C_60_ introduction into the zeolite led to observable conductivity under ambient conditions. In the presence of ethylene, these composites exhibited changes in conductivity. For recovery of the device to stable baseline values, evacuation was necessary, making it impractical for sensor use. Also, initial introduction of ethylene led to an abrupt current increase, explained as arising from temperature increase from heat of adsorption of ethylene into the zeolite.

### 5.2. Optical Measurements

All of the other reported examples of zeolite host-guest sensors are optical in nature, in which changes of spectroscopic properties of dye molecules held in zeolite cages upon interaction with analytes were used as the transduction mechanism. The two analytes that have been examined are humidity and oxygen.

Protonation/deprotonation of methylene blue in H-mordenite changes as a function of humidity and manifests itself as a change in the diffuse reflectance spectra of the composite, which has been exploited as a sensor for humidity in the 9–92% range [48,49]. Two absorption bands were distinguished, the 650 nm band due to monomeric methylene blue and 745 nm band due to the protonated methylene blue, with the 650 nm showing up in the presence of humidity and disappearing upon dehydration to form the 745 nm band.

Change in the diffuse reflectance spectra of the solvatochromic Nile red dye in zeolite NaY led to a sensor with detection limits of 200 ppm humidity and response times of ∼4 min. The discrimination against hexane was proposed as arising from its poor adsorption on the hydrophilic zeolite Y [50].

For oxygen sensing, 2-hydroxymethyl anthraquinone in zeolite NaY provides a convenient visual probe because of color change with oxygen [51]. This sensor is limited to a single use because the dihydroxy compound is converted irreversibly to the corresponding quinone, but has potential applications in the food industry.

**Figure 13.**
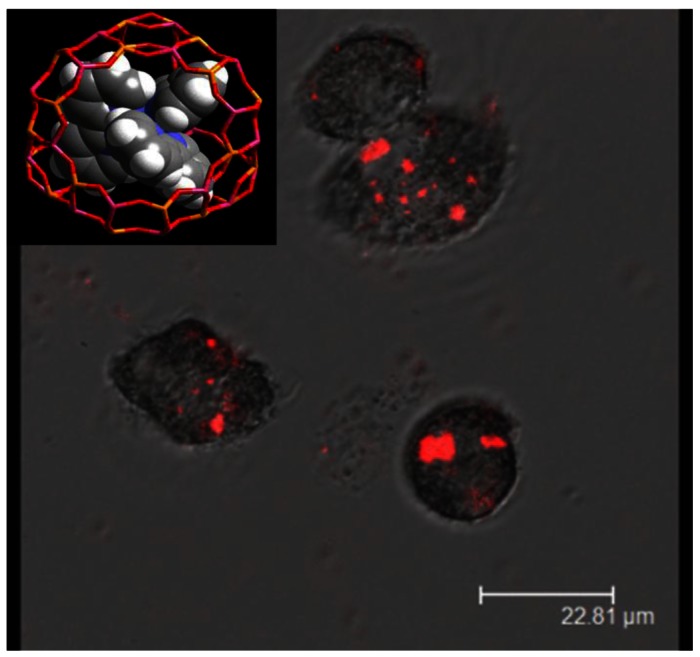
Fluorescence images of macrophage cells with tris-bipyridine ruthenium dye loaded zeolite Y (inset) (adapted from Reference [52]).

There are several reports exploiting the quenching of fluorescence of tris-bipyridine ruthenium held in zeolite cages as a measure of oxygen content. Dissolved oxygen as well as oxygen inside macrophage cells has been measured in this fashion [52,53]. Figure 13 shows the fluorescence (red) from zeolite particles in macrophage cells. This study showed that the oxidative burst within the macrophage can be tracked by measuring the emission from the dye inside the zeolite held in the cell.

## 6. Sensors Based on Intrazeolite Cation Conductivity

There are a number of recent reports on gas sensing exploiting the changes in conductivity of the extraframework cations in the zeolite. The manifestation of this change is measured by impedance spectroscopy, potentiometry, resistance and microwave methods.

### 6.1. Impedance Measurements

The interaction of basic molecules with intrazeolitic cations via Bronsted and Lewis acid-base reactions has been examined. Driven by the need for detecting ammonia used for NO_x_ reduction in the latest generation of lean burn engines, an impedance sensor using H-ZSM-5 has been reported [54].

The role of zeolite acidity and the zeolite architecture upon detection of basic molecules (water, acetonitrile, trimethylamine) using H-forms of ZSM-5, mordenite and zeolite beta has been reported [55]. For molecules smaller (<0.4 nm) than the pore diameter of the zeolites (water, acetonitrile and ammonia) the resistance calculated from the impedance spectra correlated well with the proton affinity of the base. With the larger molecules, the response was smaller. These experiments were carried out with 300 ppm of the analyte at 400 °C. The role of the zeolite architecture in influencing sensing was clear from the studies. The lower sensitivity of pyridine as compared to ammonia was correlated with the smaller size of ammonia that then reacts to form the NH_4_^+^ ion. In the case of pyridine (0.60 nm) on zeolite beta and ZSM-5, the lower sensitivity in ZSM-5 was correlated with the lower mobility of pyridinium ions (factor of 5) in MFI (pore diameter 0.51–0.56 nm) as compared to zeolite beta (0.56–0.77 nm).

**Figure 14.**
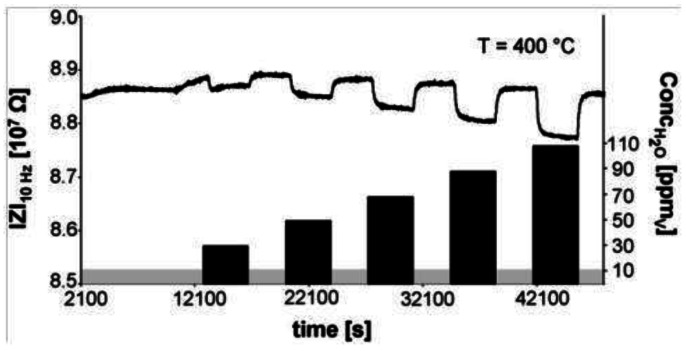
Impedance of H-ZSM-5 type zeolite as a function of water concentration at 400 °C under hydrogen environment, the bar chart is an indication of the water concentration used for the various experiments (right y-axis) (adapted from Reference [56]).

Using acid-base chemistry, a humidity sensor using H-ZSM-5 capable of operation at temperatures of 600 °C in H_2_ atmosphere has been reported [56]. The excellent thermal stability of zeolites and its inertness in reducing environments (e.g., H_2_) was exploited in this study. Typically, metal oxide or polymer based sensors designed for humidity detection will not function in reducing environment or at high temperature. Figure 14 shows the performance of the H-ZSM-5 in H_2_ at 400 °C in the presence of ppm levels of water (measurement frequency is 10 Hz). The decrease in impedance arises since the presence of water lowers the activation energy for proton motion within the zeolite.

**Figure 15.**
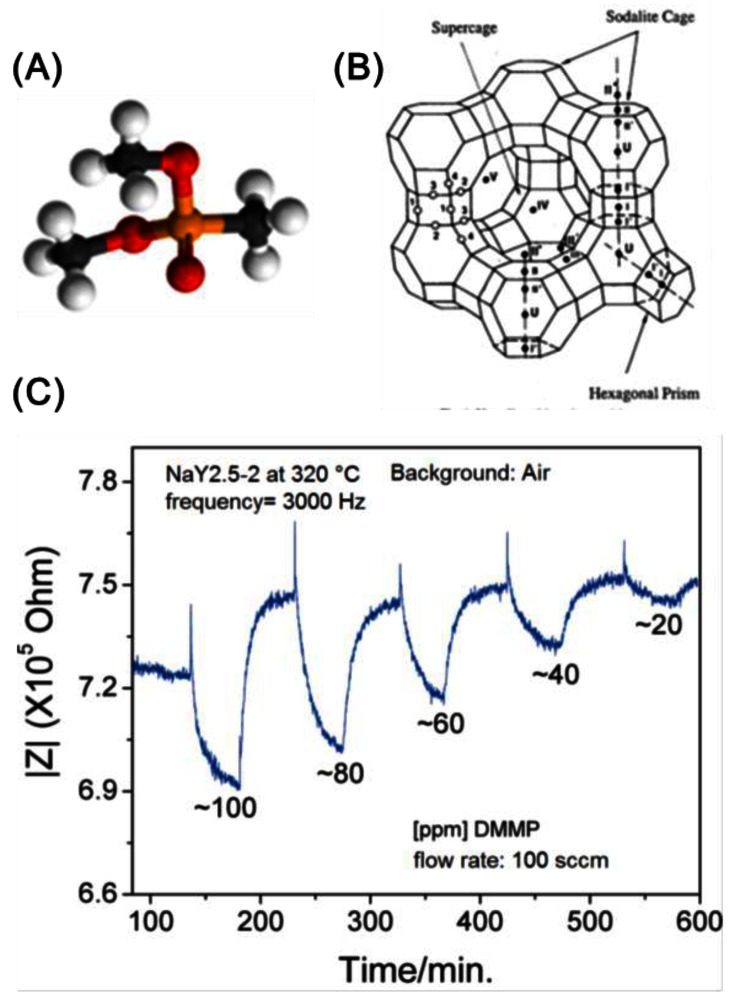
(**A**) Model of dimethylmethylphosphonate (DMMP); (**B**) Zeolite Y supercage with cation positions; (**C**) Change in impedance of Na-zeolite Y with varying concentrations of DMMP (frequency 3,000 Hz) (adapted from Reference [57]).

Interaction of dimethylmethylphosphonate (DMMP, Figure 15(A)) with monovalent cations in zeolite Y (Figure 15(B)) at 300 °C led to changes in the impedance spectra [57]. DMMP is often used as a surrogate for nerve agents. The decrease in the resistance was proposed to be due to the DMMP facilitating the cation motion (optimal results were obtained for Na^+^). Figure 15C shows the impedance at a frequency of 3,000 Hz with change in DMMP concentration.

The other group of molecules that has been extensively studied are hydrocarbons [58–60]. Impedance spectra of a device made by coating Cr_2_O_3_ on top of an ion-conducting Pt-doped ZSM-5 exhibited several semicircles, with a hydrocarbon dependent semicircle in the medium frequency range [58] (though later studies from the same group showed that it is the low frequency part that exhibits the increase in the presence of the hydrocarbons) [59,61]. Measurements at a fixed frequency in the hydrocarbon sensitive region exhibited high sensitivity to different hydrocarbons and minimal interference to CO and H_2_, but with interference to NH_3_. The proposed mechanism considered the interface between the Cr_2_O_3_/zeolite to be important rather than a filter effect from the zeolite or the semiconducting properties of Cr_2_O_3_. This interfacial effect between Cr_2_O_3_ and Pt-ZSM-5 (on the electrodes) that leads to the selective sensing of propane and propene is a novel observation. Later studies showed that the effect was not due to Cr_2_O_3_ on the zeolite layer, but Cr_2_O_3_ on the interdigitated Au electrodes produced during the photolithography process. A lower cost fabrication technology for the Cr_2_O_3_/zeolite sensor has also been proposed. A recent paper examined the mechanism at the Cr_2_O_3_/zeolite interface more carefully, and concluded that the hydrocarbon alters the charge carrier density in the Cr_2_O_3_ layer, which leads to the observed impedance changes [60]. In order to observe the response to hydrocarbon, the design has to include electrode-Cr_2_O_3_ – zeolite – Cr_2_O_3_ – electrode, or electrode – zeolite – Cr_2_O_3_ – zeolite-electrode. For samples prepared with Cr_2_O_3_ bridging the two electrodes, the unique sensitivity to hydrocarbon was not observed. Modeling studies indicated that the reaction of hydrocarbons with the Cr_2_O_3_ alters the intrinsic carrier density, which alters the depletion zone within the zeolite and results in the observed impedance changes at low frequencies. The generality of this phenomenon was demonstrated with other semiconducting oxides.

An alternative explanation has also been proposed to explain the impedance changes of the zeolite/chromia device in the presence of hydrocarbons [61]. Sorption of gas within the zeolite pores is proposed to alter the electrochemical potential at the zeolite surface. This potential leads to Na^+^ insertion from the zeolite into the chromia resulting in formation of sodium chromate at the interface, that leads to the observed impedance changes. It is difficult to decide which is the right mechanism without more experiments. However, if the formation of sodium chromate at the zeolite/chromia interface can be proven by careful electron microscopy studies, it would lend support to the second mechanism. Also, it is unclear to us how the second mechanism can explain the lack of interferences to other gases that can also adsorb within the zeolite, unless it is the reaction products of the hydrocarbon oxidation that are playing the key role, as has been alluded [61]. So experiments with water and maybe CO_2_ may also be relevant in explaining the sensor mechanism.

### 6.2. Potentiometric Measurements

Zeolites have also been examined as electrolytes for gas sensing, in particular hydrocarbons [62–64]. Using the cell Au/Na_2_CO_3_, BaCO_3_/zeolite/Au, the voltage exhibited a linear response to changes in logarithm of the propane partial pressure at 400 °C. These potentiometric sensors were based on the difference in Na^+^ activity between a reference (Na_2_CO_3_) and Na^+^ inside the zeolite. ZSM-5 was examined as the zeolite, and optimum sensitivity to propane was obtained with high Si/Al ratio of the framework. Interaction of propane with intrazeolitic Na^+^ altered its activity and resulted in the observed voltage. The zeolite thin films exhibited faster response times as compared to pellets.

### 6.3. Capacitance Measurements

**Figure 16.**
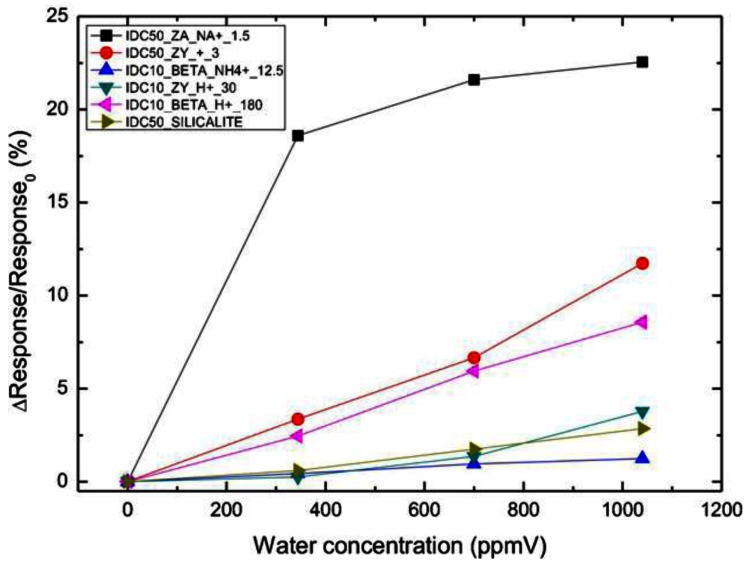
The performance of humidity sensors based on capacitance measurement of various zeolites coated on interdigitated electrodes at room temperature (adapted from Reference [65]).

Zeolite films of differing Si/Al ratios were coated on interdigitated electrodes (10–50 μm spacing) and these devices were responsive to humidity, zeolites of higher Al content exhibiting the highest sensitivity [65]. In order to promote ambient condition humidity measurements, capacitance of the zeolite rather than impedance was found to be more practical. Figure 16 shows the response based on capacitance for a series of zeolite frameworks, with the hydrophilic zeolite A (IDC 50-ZA-Na) being the most sensitive.

### 6.4. Conductivity Measurements

The sensitivity for ethanol on H-ZSM-5, zeolite Y and mordenite exhibited a decrease with increasing Si/Al ratio of the framework [66]. The mechanism of the conductivity change is proposed to arise from interaction of the ethanol with the extraframework cations.

### 6.5. Microwave Measurements

**Figure 17.**
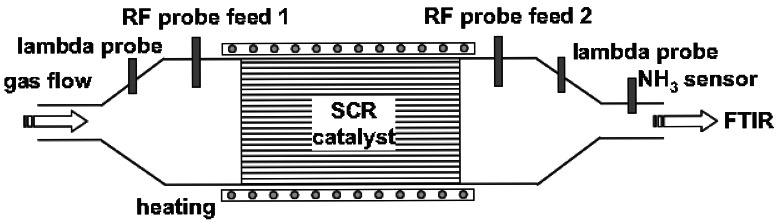
Microwave-based measurement for measuring ammonia loading for selective catalyst reduction (SCR) catalysts (adapted from Reference [67]).

Zeolite-based selective reduction catalysts (SCR) are being used for reducing NO_x_ emissions via reaction with NH_3_ in heavy duty diesel trucks. Of considerable interest is the NH_3_ loading of the catalysts. It has been demonstrated that a microwave cavity perturbation method in which electromagnetic waves at specific frequency are reflected by the catalyst and subsequently measured provide a method to estimate the stored ammonia [67]. The basis of this device is that the metallic catalyst housing (Figure 17) is a cylindrical waveguide for microwaves.

As the ammonia loading of the zeolite changes, the electrical properties of the catalyst change and is reflected in a change in the propagation of electromagnetic waves. Cross sensitivity to water was minimized by choice of the resonant frequency peak.

## 7. Conclusions

Quality and practicality of a sensor for a specific application is determined by its selectivity and sensitivity for the particular analyte. Long-term stability as well as reproducible fabrication of sensors is also critical, and often not satisfactorily reported in the literature. Other relevant aspects include operational conditions such as temperature, power requirements, footprint and ease of use. Based on the examples discussed in this review, most sensors were developed to serve the environmental industry and for optimizing combustion processes and only a few examples are found for the biomedical and food and agriculture industries. The impact of zeolites as physical and chemical filters has primarily been on enhancing selectivity, especially in the case of electrochemical sensors, which suffer often from poor selectivity. Considering the novel adsorption, diffusion and catalytic properties of zeolites, the zeolite as a selective filter is going to continue to find new applications. Newer sensing paradigms with zeolites have included chemical transformations which result in products that provide higher sensitivity than the analyte itself. This can be distinguished from sensor amplification due to preconcentration of analyte from the enhanced adsorptive property of the zeolite. With development of new microporous frameworks with novel compositions, signal enhancement will be an important feature for future applications. High throughput through very thin zeolitic films (nanometer dimensions) will improve response and recovery times. Oriented films also hold promise of rapid response. Interfacial effects in zeolite-based composites for improving sensor performance are another area that will lead to novel sensing effects. Nano sized zeolites have the potential to be incorporated into polymer packaging and can act as visual sensors, primarily for the food industry. Appropriate dye molecules need to be discovered. Intrazeolite cation mobility continues to hold promise for new applications, it is also one of the few examples that have led to commercial devices, e.g., for ammonia slip sensors. More commercial successes using zeolite incorporated sensors will accelerate development of this field. The high thermal and environmental stability of zeolites is in its favor for industrial applications. Zeolites also lends itself well for miniaturization and novel deposition and zeolite self-assembly techniques being developed will impact sensor development. Stabilization of metals, magnetic oxides, quantum dots in the nanoconfined spaces of zeolites provides opportunities for new sensors paradigms with optical or magnetic transduction methods. In summary, there appears to be considerable promise for zeolites to influence gas sensing through a variety of novel pathways, many of which have been articulated in this review article.
